# Clonal Evolution of Endometriosis to Peritoneal Endometrioid Carcinoma via *PTEN* Biallelic Inactivation: A Case Report With Integrated Genomic and Pathological Profiling

**DOI:** 10.1200/PO-26-00056

**Published:** 2026-06-08

**Authors:** Takeo Shibata, Emi Takata, Akihiro Shioya, Sohsuke Yamada, Hiroki Ura, Yo Niida, Kazuo Yasumoto, Masahiro Takakura

**Affiliations:** ^1^ Department of Obstetrics and Gynecology, Kanazawa Medical University, Uchinada, Japan; ^2^ Department of Pathology and Laboratory Medicine, Kanazawa Medical University, Uchinada, Japan; ^3^ Division of Genomic Medicine, Department of Advanced Medicine, Medical Research Institute, Kanazawa Medical University, Uchinada, Japan; ^4^ Department of Medical Oncology, Kanazawa Medical University, Uchinada, Japan

## Introduction

Endometriosis is a common gynecological disorder, yet its potential for malignant transformation into endometrioid or clear cell carcinoma represents a significant clinical concern.^1^ Recent landmark studies have revolutionized our understanding of the genomic landscape of nonmalignant endometriotic tissues.^2^ Anglesio et al^3^ reported that approximately 79% of patients with deep infiltrating endometriosis harbor somatic mutations, with 26% of lesions carrying known cancer driver mutations such as *ARID1A*, *PIK3CA*, and *KRAS*. Notably, these mutations appear to be confined to the epithelial compartment, even in lesions with virtually no risk of malignant transformation. This paradigm suggests that although cancer-associated mutations are frequently present in the endometriosis genome, only a subset of these clones eventually undergoes malignant transformation.

Although *PTEN* inactivation is a well-established driver in endometrioid malignancies, capturing the precise longitudinal moment of its biallelic loss during human carcinogenesis is extremely rare. Most previous genomic studies have focused on synchronous lesions, which provide only a snapshot of the disease at a single time point. In contrast, our case leverages a unique temporal archive by comparing current neoplastic tissues with the patient's archived normal uterine tissue from 7 years prior.

Primary peritoneal endometrioid carcinoma arising from extraovarian endometriosis is an exceedingly rare entity.^4^ A critical challenge in understanding its organogenesis is capturing the temporal and spatial continuum of this malignant transformation. Although clonal relationships between endometriosis and synchronous carcinomas have been suggested, direct genomic evidence tracking the longitudinal progression from a patient's historical normal baseline to a subsequent malignancy is scarce.

In this report, we present a unique case of primary peritoneal endometrioid carcinoma where the transition from benign endometriosis and atypical endometriosis to invasive cancer was histopathologically and genetically documented. By performing comprehensive genomic profiling (CGP) panel and whole-exome sequencing (WES) on the sequential components, we quantitatively track the clonal expansion driven by *PTEN* two-hit inactivation. Our findings highlight that the biallelic loss of *PTEN* is acquired as early as the atypical endometriosis stage, providing a high-resolution molecular basis for the organogenesis of endometriosis-associated peritoneal carcinoma.

## Case Description

### Ethical Considerations

Institutional review board approval was obtained for this study (Approval No. I193). The patient provided written informed consent for the publication of this case report.

### Clinical Course and Pathological Findings

A woman in her late 40s presented with worsening lower abdominal pain. Her medical history was significant for a total hysterectomy and left salpingo-oophorectomy performed 7 years earlier due to left ovarian endometriosis and adenomyosis. Magnetic resonance imaging revealed an enlarged right ovarian endometriotic cyst, a solid retroperitoneal tumor, and a swollen left external iliac lymph node, all of which were subsequently resected.

**FIG 1.**
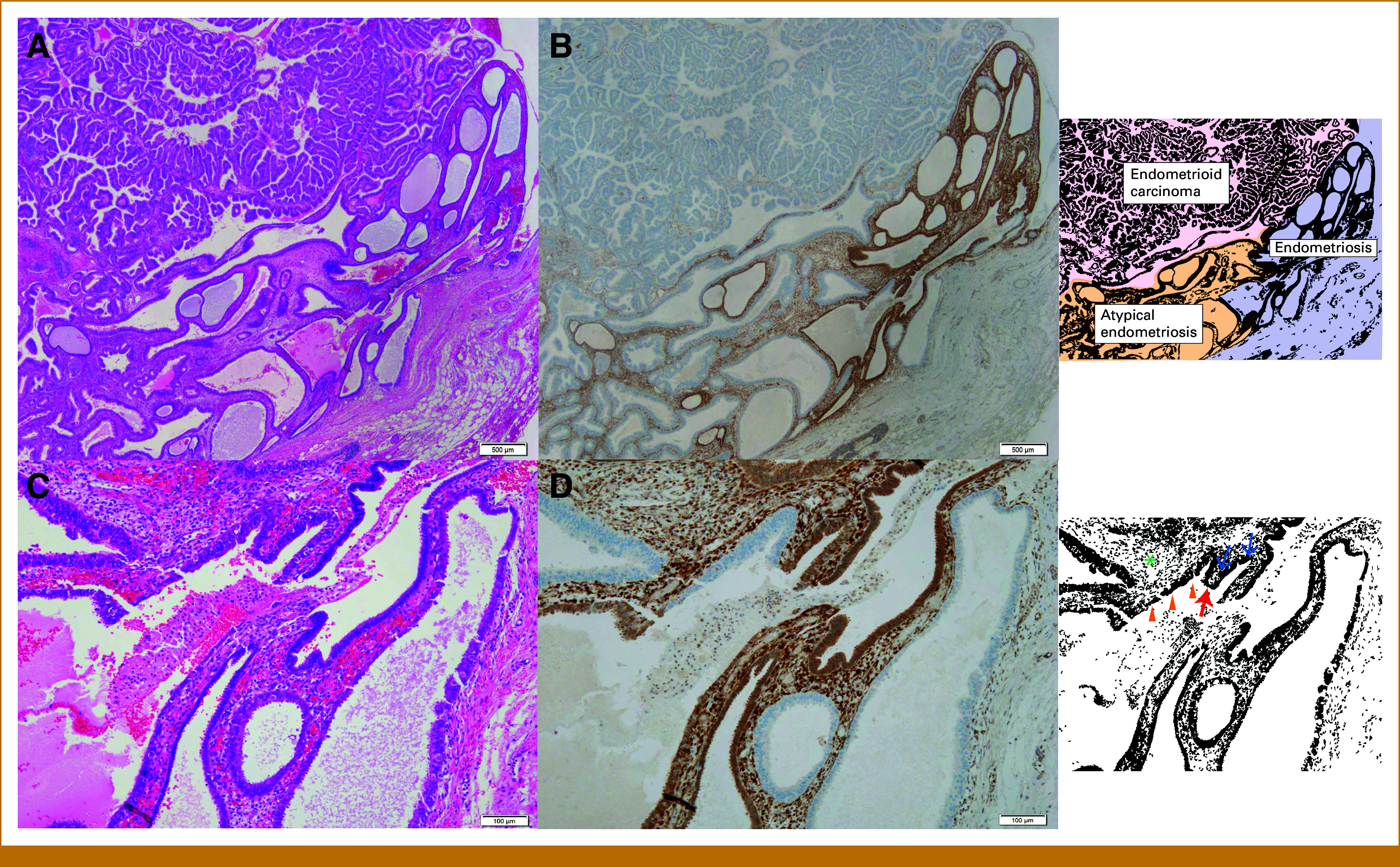
Pathological and immunohistochemical findings in the left external iliac lymph node. (A) H&E staining shows the coexistence of endometrioid carcinoma, atypical endometriosis, and benign endometriosis within the external iliac lymph node. (B) Immunohistochemical staining for PTEN reveals a distinct loss of expression in both the carcinoma and atypical endometriosis areas, whereas expression is maintained in the benign endometriosis and stroma. (C) High-magnification view of the transition zone between benign endometriosis and atypical endometriosis (H&E). (D) High-magnification view of PTEN staining in the same transition zone. The right panels show schematic diagrams of the corresponding areas. In the upper schematic, pink, yellow, and blue areas represent endometrioid carcinoma, atypical endometriosis, and benign endometriosis, respectively. In the lower schematic, yellow arrowheads indicate atypical endometriosis, and blue arrows point to benign endometriosis. The red arrow highlights the epithelial junction where the transition from PTEN-positive benign endometriosis to PTEN-deficient atypical endometriosis occurs. Green asterisks (*) indicate PTEN-positive stromal cells, serving as an internal positive control. Note that the biallelic loss of *PTEN* originates at the stage of atypical endometriosis and is sustained throughout the progression to endometrioid carcinoma. (Original magnifications: A, B = 20×; C, D = 100×. Scale bars: A, B = 500 μm; C, D = 100 μm). H&E, hematoxylin and eosin.

Histopathological examination of the resected left external iliac lymph node revealed a striking morphological continuum. The majority of the tumor mass consisted of well-differentiated endometrioid carcinoma, characterized by atypical endometrial epithelial cells showing prominent papillary growth with scant stroma. Notably, in close proximity to the malignant areas, both benign endometriosis with active cellular proliferation and atypical endometriosis (borderline tumor) were identified in direct continuity with the invasive carcinoma. This architectural transition from benign to atypical, and finally to malignant components, provided definitive evidence of the stepwise organogenesis of the tumor. Consequently, the final pathological diagnosis was established as well-differentiated endometrioid carcinoma arising in endometriosis of the retroperitoneum (Figs 1A and 1B).

Genomic testing identified the patient as having *BRCA1/2* wild-type status (BRACAnalysis) and homologous recombination-proficient (HRP) status (myChoice HRD test). On the basis of these findings, which indicated that the patient was not a candidate for PARP inhibitors, adjuvant chemotherapy consisting of six cycles of paclitaxel, carboplatin, and bevacizumab (TC+Bev) was administered, followed by bevacizumab maintenance therapy. The patient remains in complete remission at the 3-year postoperative follow-up.

### Molecular Profiling and Quantitative Variant Allele Frequency Analysis

**FIG 2.**
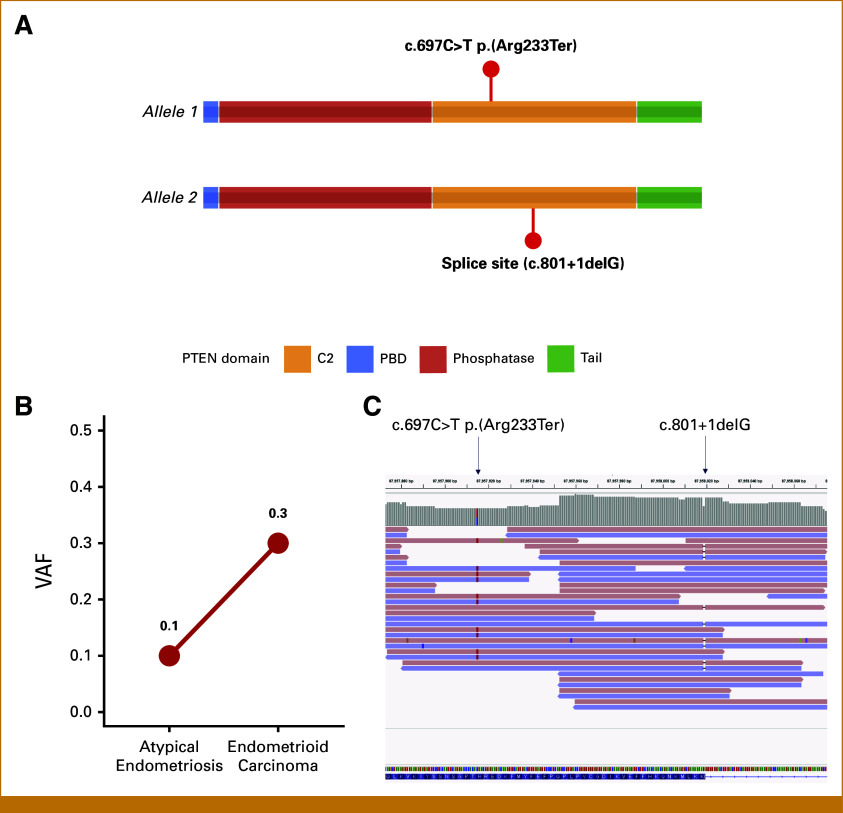
Genomic analysis and clonal evolution of *PTEN* mutations. (A) Biallelic inactivation of *PTEN*. The lollipop plot illustrates the two-hit mutations identified in the *PTEN* gene: a nonsense mutation [PTEN(NM_000314.8):c.697C>T p.(Arg233Ter)] and a splice-site mutation [PTEN(NM_000314.8):c.801+1del p.?]. Both mutations are located within or adjacent to the C2 domain, which is critical for the membrane-binding and tumor-suppressive function of the PTEN protein. The representation shows that the mutations occur on different alleles (allele 1 and allele 2). (B) Clonal progression of *PTEN*-deficient cells. The graph demonstrates the transition of VAFs from the precursor lesion to the invasive carcinoma. The VAFs of the *PTEN* mutations increased from approximately 0.1 in the atypical endometriosis stage to 0.3 in the endometrioid carcinoma, reflecting the clonal expansion of these driver alterations during malignant transformation. (C) IGV snapshots. Representative IGV images confirm that the c.697C>T and c.801+1delG mutations are mutually exclusive (located on distinct sequencing reads). This physical evidence of *trans*-configuration at the single-molecule level provides definitive proof of biallelic *PTEN* inactivation (two-hit). IGV, Integrated Genomics Viewer; PBD, PIP2 binding domain; VAF, variant allele frequency.

To investigate the molecular mechanisms of this malignant transformation, we used a strategic genomic approach using two distinct platforms. Somatic mutations were primarily analyzed using the TruSight Oncology 500 (TSO500) platform, a targeted next-generation sequencing (NGS) assay that deep-sequences 523 cancer-related genes. To further evaluate the clonal evolution and quantitative shift of these mutations, variant allele frequency (VAF) was calculated based on WES data obtained via the SureSelect XT All Exon V6 (Agilent Technologies, Santa Clara, CA) platform. We analyzed three sequential samples: archived normal uterine endometrium from the surgery 7 years prior, the current atypical endometriosis, and the invasive carcinoma. After manual inspection of the sequencing reads using the Integrated Genomics Viewer (IGV), we identified 13 high-confidence somatic variants shared between the atypical endometriosis and the carcinoma, all of which were entirely absent in the archived normal uterine tissue from 7 years prior as a baseline. These shared variants included: *IBA57* (c.641G>A), *OBSCN* (c.3443C>G), *HIST3H2A* (c.203G>A), *MUC6* (c.6068_6069delCAinsAG), *CTCF* (c.610dupA), *CDH1* (c.683delA), *MYH14* (c.5510T>C), *UCP1* (c.127G>A), *PIK3R1* (c.1145_1147delAAA), *TMEM173* (c.380C>T), and *PIK3R2* (c.599-1G>C). Notably, *PTEN* exhibited biallelic inactivation through compound heterozygous alterations: a missense mutation (c.697C>T) identified by the WES and a splice-site deletion (c.801+1delG) detected by the TSO500 panel. Quantitative VAF analysis was performed using the WES data, using the archived normal uterine tissue from 7 years prior as the somatic baseline. Compared with this mutation-free baseline, the VAF for *PTEN* mutations was approximately 0.1 in the atypical endometriosis lesion, which subsequently increased to 0.3 in the carcinoma component, reflecting significant clonal enrichment during the malignant progression (Fig 2).

### Protein Expression and Functional Correlation

To validate the functional impact of the identified biallelic *PTEN* mutations, immunohistochemistry (IHC) was performed. In the endometriosis lesions, PTEN protein expression was clearly retained in the glandular epithelium. In contrast, both the atypical endometriosis and the carcinoma components exhibited a complete loss of PTEN expression in the epithelial cells. Notably, the surrounding normal stromal cells showed strong and diffuse PTEN staining, serving as a reliable internal control (Figs 1C and 1D). This localized protein-level inactivation correlates precisely with the genomic finding of two-hit alterations, confirming that PTEN functional loss was already established at the pre-invasive stage.

## Discussion

This report presents a rare case of primary peritoneal endometrioid carcinoma, likely arising from a background of peritoneal endometriosis, in which genomic and immunohistochemical findings were longitudinally tracked across two distinct stages: atypical endometriosis and invasive carcinoma. WES and CGP using the TruSight Oncology 500 platform identified 13 shared mutations between the atypical endometriosis and the carcinoma components. Notably, the biallelic inactivation (two-hit) of *PTEN* was already completed at the stage of atypical endometriosis—before the morphological diagnosis of carcinoma—with a VAF reaching 0.1. These findings suggest that genomic priming for malignancy was significantly advanced well before the acquisition of an invasive phenotype.

The inactivation of *PTEN* is widely recognized as a foundational early event in endometriosis-associated ovarian cancer.^5^ Established literature, notably the work by Obata et al,^6^ has emphasized that loss of heterozygosity (LOH) is a hallmark of *PTEN* inactivation during the initial stages of ovarian carcinogenesis. However, our high-resolution NGS analysis revealed that the second hit in this case was not a chromosomal loss, but rather a distinct second point mutation in a *trans*-configuration. Specifically, IGV analysis demonstrated that the c.697C>T and splice-site (c.801+1delG) mutations were located on separate alleles, providing definitive evidence of the complete functional loss of the PTEN protein. This biallelic inactivation was further validated by IHC, which visually confirmed the total absence of PTEN expression in both atypical endometriotic and malignant lesions. Although Anglesio et al^3^ and Suda et al^2^ found cancer-associated mutations in benign endometriosis, our study demonstrates that biallelic inactivation during the atypical stage drives decisive clonal expansion. The correlation between this genomic two-hit event and proteomic loss confirms that *PTEN* deficiency was established well before morphological malignancy. This underscores the utility of longitudinal genomic tracking in dissecting the stepwise progression from benign and atypical endometriosis to invasive carcinoma.

The C2 domain, where mutations were concentrated in this case, is essential for recruiting PTEN to the cell membrane. Its disruption impairs inhibitory control over the PI3K/Akt/mTOR pathway, even with a conserved phosphatase domain. This pathway represents a critical therapeutic target. Clinical trials like CAPItello-292 are evaluating the Akt inhibitor capivasertib for patients with breast cancer with *PTEN* loss or *PIK3CA* mutations.^7^ Such biomarker-driven therapies hold promise for endometrioid carcinomas with profound *PTEN* inactivation. Furthermore, *PTEN* deficiency is thought to upregulate vascular endothelial growth factor via HIF-1α stabilization through the activated PI3K/Akt/mTOR pathway.^8^ Thus, this genomic background provides a biological rationale for bevacizumab.

Several limitations of this study should be acknowledged. First, as a single case report, our findings regarding the timing of *PTEN* inactivation and clonal evolution are specific to this patient and require large-scale validation. Second, the VAFs did not perfectly correlate with microscopic tumor density due to the nature of manual macrodissection. Since VAF measures the proportion of mutant DNA molecules rather than a visual area ratio, it reflects the actual tumor DNA fraction within bulk samples. In the atypical endometriosis lesion, a VAF of 0.1 aligns with the approximately 20% epithelial fraction, where PTEN-deficient glands were intermingled with stroma and lymphocytes at a 1:4 ratio. Conversely, although the carcinoma showed approximately 70% tumor density in specific fields, the VAF of 0.3 reflects DNA from bulk samples that inevitably included non-neoplastic stroma and inflammatory infiltrates. Despite this dilution, the complete loss of PTEN expression in epithelial cells provides definitive spatial evidence of biallelic functional inactivation.

## Conclusion

Our findings suggest a potential molecular pathway where underlying endometriotic lesions, including atypical components, serve as a reservoir for genomic evolution in rare primary peritoneal endometrioid carcinomas. The transformation of a radiologically occult lesion into a carcinoma with complex genomic alterations over 7 years illustrates the progressive nature of this entity. Notably, biallelic *PTEN* inactivation at the preinvasive stage indicates that genomic acceleration may precede morphological malignancy. Although further investigation in larger cohorts is required to determine the precise timing of these changes, elucidating such molecular shifts could eventually contribute to early diagnostic tools and personalized therapeutic strategies for endometriosis-associated malignancies.

## Data Availability

A data sharing statement provided by the authors is available with this article at DOI https://doi.org/10.1200/PO-25-00056.
